# Single Camera-Based Dual-Channel Near-Infrared Fluorescence Imaging system

**DOI:** 10.3390/s22249758

**Published:** 2022-12-13

**Authors:** Janghoon Choi, Jun-Geun Shin, Yoon-Oh Tak, Youngseok Seo, Jonghyun Eom

**Affiliations:** 1 Intelligent Photonic IoT Research Center, Korea Photonics Technology Institute, Gwangju 61007, Republic of Korea; 2 Department of Biomedical Science & Engineering, Gwangju Institute of Science and Technology, Gwangju 61005, Republic of Korea; 3 Optical Precision Measurement Research Center, Korea Photonics Technology Institute, Gwangju 61007, Republic of Korea; 4 WONTECH Co., Ltd., Daejeon 34028, Republic of Korea

**Keywords:** near-infrared fluorescence imaging system, dual-channel fluorescence imaging, real-time imaging, indocyanine green, methylene blue

## Abstract

In this study, we propose a single camera-based dual-channel near-infrared (NIR) fluorescence imaging system that produces color and dual-channel NIR fluorescence images in real time. To simultaneously acquire color and dual-channel NIR fluorescence images of two fluorescent agents, three cameras and additional optical parts are generally used. As a result, the volume of the image acquisition unit increases, interfering with movements during surgical procedures and increasing production costs. In the system herein proposed, instead of using three cameras, we set a single camera equipped with two image sensors that can simultaneously acquire color and single-channel NIR fluorescence images, thus reducing the volume of the image acquisition unit. The single-channel NIR fluorescence images were time-divided into two channels by synchronizing the camera and two excitation lasers, and the noise caused by the crosstalk effect between the two fluorescent agents was removed through image processing. To evaluate the performance of the system, experiments were conducted for the two fluorescent agents to measure the sensitivity, crosstalk effect, and signal-to-background ratio. The compactness of the resulting image acquisition unit alleviates the inconvenient movement obstruction of previous devices during clinical and animal surgery and reduces the complexity and costs of the manufacturing process, which may facilitate the dissemination of this type of system.

## 1. Introduction

Medical imaging technology has been continuously developing for the diagnosis and treatment of cancer [1]. Imaging techniques such as X-ray imaging, computed tomography (CT), magnetic resonance imaging (MRI), positron emission tomography (PET), single-photon emission computed tomography (SPECT), and ultrasound imaging are helpful in the early-stage detection of various cancers [2,3,4]. Although these imaging devices are used to diagnose cancer, they have limitations in intraoperative image-guided surgery. It is difficult to show images in real time because of the continuous exposure to radiation or magnetic fields as well as slow data processing speed. Ultrasound imaging equipment has limitations depending on the surgical site and method because the detector must be in direct contact with the affected part. The near-infrared (NIR) fluorescence imaging device acquires fluorescence images while being far from the affected area after injecting an FDA-approved fluorescent contrast agent, such as indocyanine green (ICG) or methylene blue (MB), into the patient. It is suitable for intraoperative image-guided surgery because it can detect lesions or specific tissues that cannot be identified with the naked eye using a fluorescent contrast agent, and it provides intuitive information by matching the visible-light image and the fluorescent image in real time [5].

Owing to these advantages, clinical surgery with ICG is used in sentinel lymph node mapping for various cancers such as breast cancer [6,7,8], vulvar cancer [9], bladder cancer [10], and cutaneous melanoma [11]. Additionally, it is used to identify and protect the parathyroid gland during thyroidectomy [12] and to identify and remove parathyroid adenomas during parathyroidectomy [13]. Moreover, it is used in intraoperative tumor detection [14,15], laparoscopic cholecystectomy [16], perforator flap reconstruction [17], and living-donor nephrectomy [18]. In particular, it is expected to replace conventional surgical methods for sentinel lymph node mapping. Traditionally, a radioactive substance was injected into the cancer to determine the location of the lymph node using a radiation detector, or blue dye was injected into the cancer to locate the lymph node with the naked eye after skin incision. However, the lymph node mapping accuracy using a fluorescent imaging device was better than when each method was used separately, and was almost similar to when both methods were used together [19]. The use of NIR fluorescence imaging devices is expected to increase further as they do not necessitate the handling of radioactive materials and ease the locating of lymph nodes using real-time images. In addition, the clinical application of MB includes imaging of breast cancer tissue during breast-conserving surgery [20], mapping of sentinel lymph nodes of breast cancer [21], identification of parathyroid adenoma [22], imaging of paraganglioma [23], and identification of the ureters [24,25]. Recent studies show the NIR fluorescence imaging technique can help the discrimination of inflammation and cancer [26,27].

Due to the great potential of the NIR fluorescence imaging system, various types of NIR fluorescence imaging systems have been developed. There are also relatively simple products that provide only handheld fluorescence images [6,7], and most systems provide one-channel NIR fluorescent images, color images, and merged images of NIR and color images [28]. In addition to open surgery imaging systems, endoscopy and laparoscopy for minimally invasive surgery have also been developed [16,29]. A new imaging system that provides both ICG and MB images simultaneously in different channels was developed [30]. The system uses three cameras for two-channel fluorescent images and one-channel color images, and several additional optical components are used to separate the visible, NIR 1, and NIR 2 bands coming from the lens group and maintain the same optical path. Therefore, the volume of the imaging system increases, which can interfere with surgical movement, and the system configuration cost also increases.

To overcome these shortcomings, we developed a compact image acquisition unit that incorporates a single camera with two built-in image sensors instead of the three cameras conventionally used. The camera and a couple of lasers that excite two different fluorescent agents were synchronized by trigger signals, and NIR fluorescent images were obtained by sequentially turning on and off the two lasers, and then converting them into two-channel NIR fluorescent images through image processing. To evaluate and optimize the performance of the system, the sensitivity, crosstalk effect, and signal-to-background (SBR) ratio were measured according to the camera exposure time and gain as well as the concentration of the two contrast agents. Subsequently, we discuss the advantages and disadvantages of the proposed system, particularly highlighting its simpler and more economical manufacturing process compared with that of systems using three cameras [30].

## 2. Materials and Methods

### 2.1. System Design

**Figure 1:**
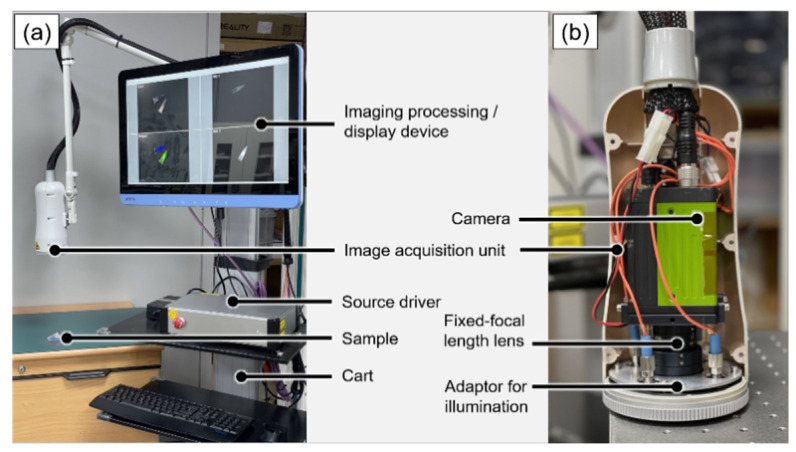
(**a**) Configuration of the proposed NIR fluorescence imaging system. (**b**) Image acquisition unit consisted of the single camera and the fixed-focal-length lens and adaptor for illumination parts.

Figure 1 shows the configuration of the proposed imaging system, which was built by adding a 680 nm light source and a trigger to the system previously developed by our group [31]. The image acquisition unit for image acquisition and illumination comprised a camera, a fixed-focal length lens, and an adapter for mounting the illumination parts. A single camera equipped with two complementary metal–oxide–semiconductor (CMOS) image sensors, capable of acquiring images in the visible and NIR bands, was used. Image signals entering a common optical path were separated into visible-light bands and NIR bands using a dichroic prism; subsequently, they were acquired by a color image sensor and an NIR image sensor, respectively. An 8.5 mm fixed-focal-length lens was used, and the field of view (FOV) was measured as 206 mm × 154 mm at a working distance of 30 cm. By fixing the adapter to the focusing part of the lens, focus adjustment was made easy, even after the image acquisition unit was packaged. A notch filter (#67-112, Edmund Optics, Barrington, NJ, USA) was installed at the center of the adapter where the image signal came in to prevent a decrease in the SBR due to the specular reflection of the 785 nm laser diode (LD). Four 3 W white LEDs (W42180; Seoul Semiconductor, Ansan, Republic of Korea) and four connecting ports of LDs for fluorescent excitation were installed on the outside of the adapter. For the excitation of the fluorescence contrast agent, a 680 nm LD 785 nm LD was used and controlled through the current driver.

**Figure 2:**
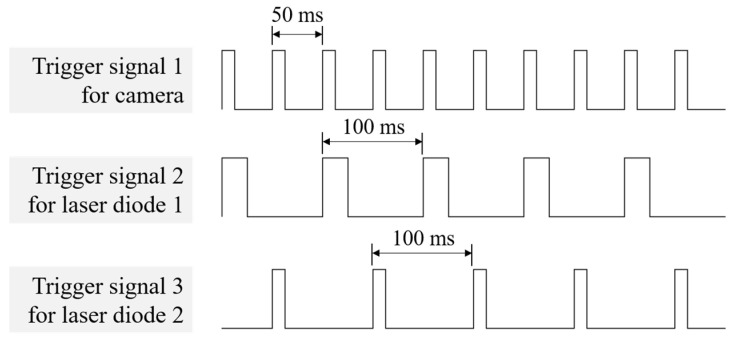
Trigger signals for synchronized operation of the camera and the two laser diodes for NIR dual-channel fluorescence imaging.

Figure 2 illustrates the trigger signals used in the system; the trigger signals were generated by the encoder trigger board (MVENC852v3, MV Solutions, Seongnam, Republic of Korea). The three trigger signals were synchronized, each operating either the camera, 680 nm LD, or 785 nm LD. The camera acquired an image according to the rising edge of the trigger signal, the LD was turned on when the trigger signal gave a positive voltage. The trigger signal interval of the camera was 50 ms, and color and fluorescence images were continuously acquired 20 times per second. The trigger signal interval of each LD was 100 ms, and the two LDs were alternately turned on and off 10 times per second.

**Figure 3:**
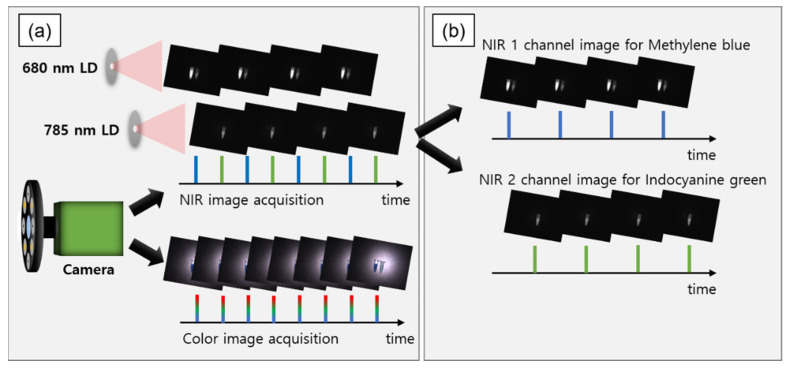
Data acquisition sequence during dual-channel NIR fluorescence imaging: (**a**) color and NIR image acquisition by the camera, with NIR images being sequentially acquired under different LDs; (**b**) division of NIR images into two channels according to the illumination by the two LDs.

The data acquisition method based on the trigger signal is shown in Figure 3. The NIR fluorescence images acquired according to the trigger signal were sequentially divided into two channels. When the 680 nm LD was turned on, NIR channel 1 acquired MB and ICG fluorescence images owing to the crosstalk effect. To remove the crosstalk effect of ICG from the NIR 1 channel image, an image processing method was applied, where the previous NIR 2 channel image was subtracted. Since there was no MB crosstalk effect under 785 nm LD illumination, only ICG fluorescence images were acquired in the NIR 2 channel.

### 2.2. System Characterization

In the developed system, the intensity of the fluorescence signal is determined by the type and concentration of the fluorescent contrast agent, the intensity of illumination, illumination time, and exposure time and gain of the camera. The exposure time is the duration that the imaging sensor of the camera is actually subjected to the light to capture an image, whereas the gain is the signal amplification of the camera, which, when increased, augments the intensity of the signal and noise. To measure the sensitivity of the system to the fluorescence contrast agent, the intensity of the fluorescence signal was analyzed in accordance with the concentrations of MB and ICG, exposure time, and gain of the camera. MB (M5528-25G, Sigma-Aldrich, Merck KGaA, Darmstadt, Germany) and ICG (RFP0815, Bioacts, Incheon, Republic of Korea) were diluted with deionized water, and each sample was stored in a 1.5 mL micro tube. The concentration of MB was 0.05, 0.1, 0.2, 0.5, 1, 2, 5, 10, 20, 50, 100, 200, 500, 1000, 2000, and 5000 μM, the concentration of ICG was 0.01, 0.02, 0.05, 0.1, 0.2, 0.5, 1, 2, 5, 10, 20, and 50 μM. The current applied to the 680 and 785 nm LDs was fixed at 2.0 A and irradiated on a fluorescent contrast agent, the camera gain was set to 1, and a sample was taken while changing the exposure time to 10, 20, 30, and 40 ms. In the NIR fluorescence images obtained under each condition, the grayscale average values of 5000 pixels in each sample area were measured.

The crosstalk effect occurred because of the overlapping bands of the absorption and emission spectra of the two fluorescent contrast agents. The ICG samples were irradiated with 680 nm LD adopted for MB excitation, and the MB samples were irradiated with 785 nm LD to analyze the intensity of the fluorescence signal due to the crosstalk effect. The current of each LD, concentration of the sample, exposure time and gain of the camera, and analysis method of the results were carried out similar to the sensitivity measurement experiment.

The background noise was measured to determine the factors affecting the SBR of the fluorescence images. Background images were acquired when the white LED was on and off and when the background was a black-anodized aluminum plate or white paper. As the exposure time and gain of the camera increased under each condition, the average value of the gray level of all pixels was calculated.

The SBR was measured according to the concentration of the sample and the exposure time and gain of the camera. MB samples at concentrations of 0.5, 5, 50, 500, and 5000 μM and ICG samples at concentrations of 0.05, 0.5, 5, and 50 μM were irradiated with 680 nm and 785 nm LD set at a current of 2.0 A. NIR fluorescence images were obtained for each sample and no sample at exposure times of 10, 20, 30, and 40 ms and gains of 1, 4, 7, 10, 13, and 16 with and without 12 W white LED illumination. In each image, the SBR was calculated by dividing the grayscale average of 5000 pixels in the sample area by the average value of 5000 pixels in the same position when there was no sample.

## 3. Results

**Figure 4:**
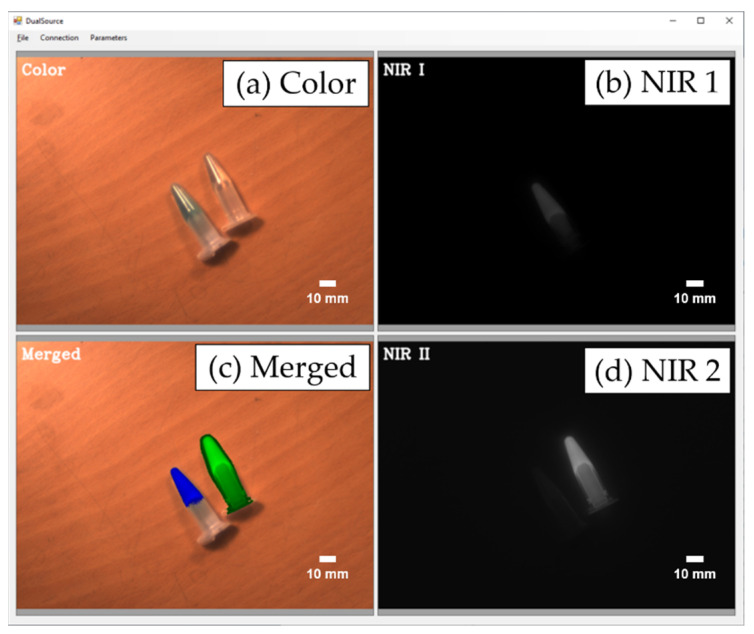
Real-time display images of methylene blue (MB) and indocyanine green (ICG). (**a**) Color image; (**b**) NIR 1 image (MB); (**c**) pseudo color merged image; (**d**) NIR 2 image (ICG).

We evaluated the feasibility of the proposed method using two fluorescent agents (ICG and MB). Figure 4 shows the images acquired by the real-time fluorescence imaging system; visible image (Figure 4a), NIR 1 image for the MB fluorescent contrast agent (Figure 4b), NIR 2 image for the ICG fluorescent contrast agent (Figure 4d), and merged image (Figure 4c) can be observed in the figure. The merged image was matched to a color image by applying the pseudo color of two NIR fluorescence images in real time. The acquired images were separated using the proposed time-division technique.

**Figure 5:**
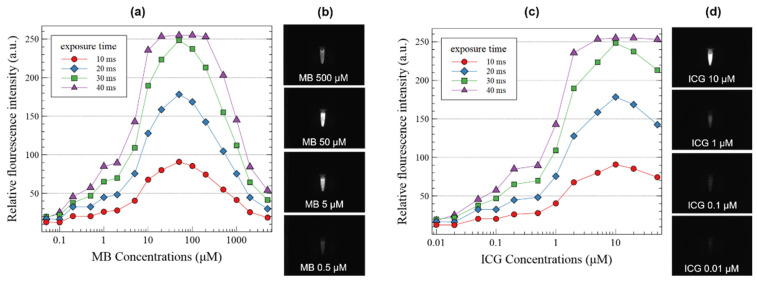
(**a**) Sensitivity of methylene blue (MB) and (**c**) indocyanine green (ICG) depending on the concentrations of the two fluorescent contrast agents and exposure time of the camera. (**b**) NIR images of MB; (**d**) NIR images of ICG.

The sensitivity to MB and ICG is shown in Figure 5. The intensity of the fluorescence image increased as the concentration of the fluorescence contrast agents and exposure time of the camera increased. Because the fluorescence molecules that are excited by the laser and emit the light increase. The exposure time of the camera, which is the time to collect the light emitted from the contrast agent, showed a linear relationship with the fluorescence image intensity. Peak values were measured when MB and ICG were 50 μM and 10 μM, respectively, and the fluorescence intensity was observed to decrease at higher concentrations due to self-absorption. The fluorescence signal increased as the exposure time increased. When the exposure time was 40 ms, the fluorescence signal saturated at MB concentrations of 20, 50, 100, and 200 μM, and the ICG concentration saturated at 5, 10, and 20 μM. For samples with high sensitivity, the exposure time of the camera or intensity of the LD should be reduced to avoid saturation.

**Figure 6:**
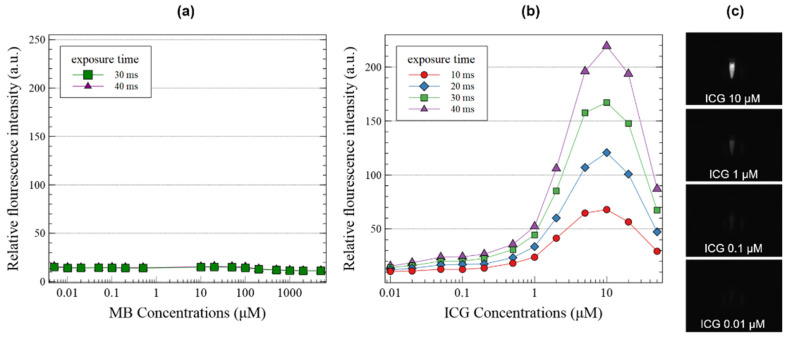
(**a**) Crosstalk effect of the methylene blue (MB) under 785 nm laser diode (LD) and (**b**) indocyanine green (ICG) under 680 nm LD depending on the concentrations of the two fluorescent contrast agents and exposure time of the camera (**c**) NIR images of ICG under 680 nm LD.

The crosstalk effect of fluorescent agents under different excitation lights was measured, and the results are shown in Figure 6. For MB, the fluorescence signal was not measured at 785 nm LD, whereas for ICG, the fluorescence signal was measured at 680 nm LD. The fluorescence signal of ICG under 680 nm LD was weaker than that under 785 nm LD; however, the response according to the concentration was almost similar.

**Figure 7:**
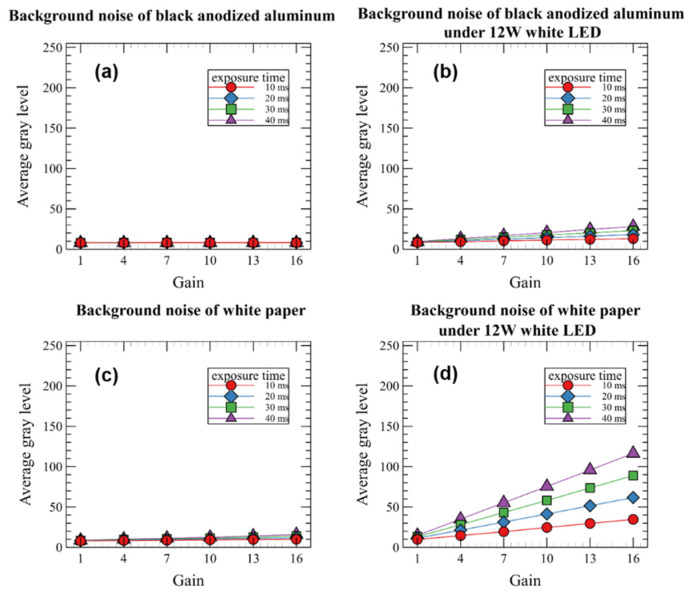
Background noise of black-anodized aluminum plate (**a**,**b**) and white paper (**c**,**d**) with and without 12 W white LED illumination according to the exposure time and gain of the camera.

Figure 7 shows the results of analyzing the grayscale average of the background image according to the type of background and the exposure time and gain of the camera. As shown in Figure 7a,c, when the white LED was off, the background noise was significantly low, even when the exposure time and gain were increased. However, as shown in Figure 7b,d, when the white LED was on, the background noise steadily increased as the exposure time and gain increased. In addition, the background noise varied greatly depending on the background, and the grayscale average of the white paper image was 1.4 to 3.5 times higher than that of the black anodized aluminum plate while the gain of the camera increased from 1 to 16, because the reflectance of white paper and black anodized aluminum in NIR bands is almost 0.7 and 0.35, respectively [32,33]. In particular, the average grayscale of the white paper background increased to 100 or more. The afore-described experiment was performed according to the on-off of the LDs; however, a significant difference was not observed.

**Figure 8:**
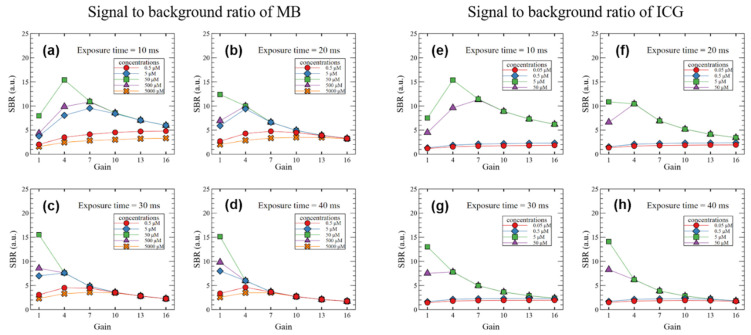
SBR of (**a**–**d**) MB and (**e**–**h**) ICG according to the concentrations of the two fluorescent contrast agents and the exposure time and gain of the camera.

The signal-to-background ratio was measured as a function of the sample concentration and the exposure time and gain of the camera, and the results are shown in Figure 8. Samples with good sensitivity showed a high SBR when the gain was 1–4 depending on the exposure time. In samples with low sensitivity, the SBR increased at a gain of 4–10 depending on the exposure time. The SBR measured when the white LED was off increased by 17–32% as the gain increased as compared to when the white LED was on.

## 4. Discussion

NIR fluorescence imaging technology, which can shorten surgery time and increase the accuracy of surgeries by imaging lesions that are difficult to distinguish with the naked eye in real time, is being applied in various surgeries. In addition, the development of a variety of fluorescent contrast agents capable of tagging specific cancers or tissues further increases the potential of this imaging system [34]. The NIR fluorescence imaging system is equipped with a function to provide high-sensitivity images of fluorescence contrast agents in real time. In general, two cameras are used for color and fluorescence images, and three cameras are used for color and two-channel fluorescence images. Additional optical components are required to match the color and fluorescence images, and to filter according to the absorption and emission bands of the contrast agents.

In this study, we developed a compact system that uses a single camera equipped with two image sensors. By controlling the camera and two LDs using synchronized trigger signals, one color and two channel NIR fluorescence images were acquired using a single camera and displayed in real time. Through sensitivity and signal-to-background experiments for system optimization, the system setting method according to the concentration of the contrast medium was presented.

The main challenge in developing this system is the acquisition of two channels of fluorescence images from one near-infrared image sensor. We acquired two-channel images by controlling the camera and two LDs with a synchronized triggering signal, acquiring images by time division, and displaying the acquired images in different channels, one frame at a time. Contrary to the notion that the crosstalk effect could be controlled by applying an appropriate threshold value, the intensity of the fluorescence image from ICG under 680 nm LD illumination was extremely strong. To solve this problem, the ICG fluorescence image expressed under 680 nm LD was removed by subtracting the NIR 2 channel image of ICG from the NIR 1 channel image of the MB. Since many factors affect the SBR of NIR fluorescence images, the concentration of the sample and the exposure time and gain of the camera must be appropriately adjusted depending on the situation. The change in the fluorescence signal according to the change in these variables was demonstrated through the experiment, and a guideline for a better image was presented to the user.

**Figure 9:**
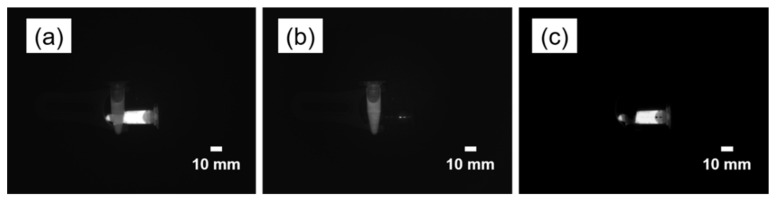
NIR Fluorescence images of crossed two contrast agents: methylene blue and indocyanine green. (**a**) NIR 1 image under 680 nm laser diode illumination and (**b**) NIR 2 image under 785 nm laser diode illumination, and (**c**) subtracted image by NIR 2 from NIR 1 image.

However, the method used to eliminate the crosstalk effect has drawbacks. When removing the ICG fluorescence signal in the situation where the MB sample is superimposed under the ICG sample, the fluorescence signal of the MB can also be removed. This is shown in Figure 9. This did not occur when the MB sample was immediately adjacent to or above the ICG sample. Another disadvantage is that one NIR image sensor is time-divided and used as two channels; therefore, there is a limitation in that the frame rate of the fluorescence image is reduced by half. However, because the adopted single camera supports up to 226 fps in an 8-bit video format, an appropriate frame rate can be secured through software optimization. However, there is a trade-off between the exposure times of the cameras. Similarly, another limitation is that the exposure time and gain values of the two channels cannot be set differently. In clinical use, if there is a large difference in the intensity of the fluorescence signal of the two channels depending on the concentration of the fluorescence contrast agent, depth of the target tissue, or lighting conditions, it may not be possible to obtain an appropriate image because of a low fluorescence signal or a saturated fluorescence signal. As a solution, the difference in sensitivity of the fluorescence signal can be adjusted by adjusting the illumination time differently by changing the trigger signal width of the two LDs.

This system has advantages such as miniaturization of the image acquisition unit, simplicity of the manufacturing method, and reduction in manufacturing costs. Since images are captured above the affected area in NIR fluorescence imaging devices, the miniaturization of the image acquisition unit owing to the use of a single camera and the compact design of the illumination parts minimizes the obstruction of movement during the operators. In addition, unlike a general system that uses three cameras, mirrors, lenses, and an additional optical system for the light alignment and illumination unit in the image acquisition unit, the developed system can be manufactured simply by sequentially connecting a single camera, a fixed-focal-length lens, and an illumination unit. In addition, because the LD can be turned on and off through the synchronized trigger signal, and the luminescence time of the LD can be adjusted according to the sensitivity, it can minimize damage to the living tissue due to light exposure compared to other systems that continuously illuminate the affected area. It can also reduce the heat generation of the system and energy consumption.

## 5. Conclusions

We developed a compact NIR fluorescence imaging system that simultaneously acquires color and dual-channel NIR fluorescence images using a single camera equipped with two image sensors and displays them in real-time. The images captured by one NIR image sensor were divided according to time into two channels by controlling the image acquisition of the camera and the illumination of the two laser diodes with synchronized trigger signals. Through experiments on the fluorescence imaging characteristics of the developed system according to the concentration of the fluorescence contrast agent and camera settings, a setting method to obtain an image with a high signal-to-background ratio was devised, and the advantages and disadvantages of the system were analyzed. The results indicate that, compared with systems using three cameras, the compact image acquisition unit proposed interferes less with the movements of the operator during surgery, and allows for a simplified and more economical manufacturing process. However, some limitations remain unresolved, such as the reduction of the exposure time of the camera and frame rate of the NIR images by 1/2, and the invisibility when MB is under ICG. In the future, we will focus on clinical applications by developing software with improved user convenience based on characteristic analysis and optimizing the system through animal studies.

## Data Availability

Not applicable.
